# Quality of reporting on randomized controlled trials of acupuncture for stroke rehabilitation

**DOI:** 10.1186/1472-6882-14-151

**Published:** 2014-05-07

**Authors:** Lixing Zhuang, Jun He, Xun Zhuang, Liming Lu

**Affiliations:** 1 Guangzhou University of Chinese Medicine, Guangzhou 510405, China; 2 Department of Acupuncture, First Affiliated Hospital, Guangzhou University of Chinese Medicine, Guangzhou 510405, China; 3 Faculty of Medical Statistics and Epidemiology, School of Public Health, Sun Yat-sen University, Guangzhou 510080, China

**Keywords:** Acupuncture, Quality of reporting, Randomized controlled trials (RCTs), Stroke rehabilitation, The CONSORT statement, STRICTA

## Abstract

**Background:**

Results from clinical studies on acupuncture for stroke rehabilitation are contradictory. The reason for the inconsistent findings especially lie in the transparency and accuracy of randomized controlled trials (RCTs) reports. This study aims to analyze the quality of reporting and its correlates in RCTs on acupuncture for stroke rehabilitation.

**Methods:**

Quality of reporting for included papers was assessed against a subset of criteria adapted from the CONSORT 2010 statement and STRICTA. An overall quality score (OQS) and a combined key methodological index score (MIS) was calculated for each trial. Then, factors associated with OQS and MIS were identified.

**Results:**

A total of 15 RCTs were included in full text. The median OQS based on the CONSORT statement and STRICTA was 8 and 12, respectively. The significant predictors for CONSORT OQS was funding source, for STRICTA was year of publication. With regard to the MIS, no variable was associated with improved methodological quality.

**Conclusions:**

Our study found that the overall quality of reporting on RCTs of acupuncture for stroke rehabilitation was general or good. But some items’ reporting was found where information was insufficient or inadequate in most studies which needed substantial improvement.

## Background

Stroke is a leading cause of death in both middle- and high-income countries, responsible for 22% of all deaths worldwide [1]. The risk factors leading to increasing stroke incidence are an aging population, dietary changes, and work-related stress, etc. In China, stroke is the second most common cause of death in cities and the third most common cause of death in rural areas [2]. Also, stroke is a major cause of disability and dependency. Poststroke disability with a high incidence (>50%) brings a heavy burden to patients and their caregivers [3]. In the United States, the total societal and healthcare costs have risen from $53.6 billion in 2004 [4] to $68.9 billion in 2009 [3]. Clearly, patients with stroke urgently need safe and effective treatments for alleviating the burden, which drives people to search for conventional treatment to improve the effect of stroke rehabilitation, such as acupuncture.

For more than 3000 years, practitioners in China have used acupuncture to treat various diseases, including the sequelae of stroke. Acupuncture is widely used to improve motor, sensation, speech and other neurological functions in patients with stroke. Compared to other conventional interventions, acupuncture is relatively simple, inexpensive and safe which has been well accepted by Chinese patients and also increasingly practiced in some Western countries [5]. Results from clinical studies on acupuncture for stroke rehabilitation, however, are contradictory. Although some reviews indicate that too little evidence exists to prove the efficacy of acupuncture for stroke patients [6-9], a series of studies suggest that this modality is an effective method for improving disabilities due to stroke [10-13]. Researchers have called the quality of these meta-analyses into question, however, and the likely potential for publication bias suggests a need for additional research on the subject. Currently, it is necessary to evaluate the quality of reporting on randomized controlled trials (RCTs) of acupuncture for stroke rehabilitation, for quality of reporting is essential for guiding journal peer-review decisions, for experts’ recommendations, and to conduct unbiased meta-analysis, as it influences our interpretation of evidence.

The Consolidated Standards for Reporting Trials (CONSORT) statement developed in 1996 [14] aimed to facilitate critical appraisal and interpretation of RCTs by authors, providing them guidance about how to improve the reporting of their trials, as well as by peer reviewers and editors, helping them identify reports with potentially biased results. The CONSORT statement has been further revised and is published as the CONSORT 2010 statement. Combined with the CONSORT statement, the acupuncture-specific Standards for Reporting Interventions in Controlled Trials of Acupuncture (STRICTA) were designed to help authors fully report the acupuncture intervention in clinical trials [15,16]. The combination of both tools helps us evaluate the completeness and transparency of RCTs reports on acupuncture for stroke rehabilitation according to their items.

However, to the best of our knowledge, there are no data about the quality of RCTs reporting in acupuncture for stroke rehabilitation. The present study aims to (i) describe the characteristics of published RCTs reports on acupuncture for stroke rehabilitation; (ii) assess the overall quality and key methodological items of published articles of RCTS on acupuncture for stroke rehabilitation; and (iii) determine factors associated with better reporting quality.

## Methods

### Search strategy

We searched the Cochrane Stroke Group Trials Register (March 2013), the Chinese Stroke Trials Register (March 2013), the Chinese Acupuncture Trials Register (March 2013) and the Trials Register of the Cochrane Complementary Medicine Field (March 2013). In addition, we searched the following bibliographic databases: MEDLINE (1966 to March 2013), EMBASE (1980 to March 2013), CINAHL (1982 to March 2013), AMED (the Allied and Complementary Medicine Database, 1985 to March 2013), the Chinese Biological Medicine Database (CBM-disc, 1979 to March 2013), China National Knowledge Infrastructure (CNKI, 1979 to March 2013), Wanfang databases (1982 to March 2013) and VIP database (1992 to March 2013). The following search terms were used in Chinese and English: poststroke, hemorrhagic, ischemic, cerebrovascular disorders, acupuncture, electro-acupuncture, needle, scalp acupuncture, auricular acupuncture, acupuncture point, meridian, acupoint and point injection, etc.

### Inclusion & exclusion criteria

#### Types of studies

RCTs comparing acupuncture with at least one control group that used placebo, sham treatment or conventional treatment in patients with subacute (one to three months since onset) or chronic stroke (over three months since onset) were included in this research. RCTs were included if invasive acupuncture was used as the sole intervention or as an adjunct to another standard treatment for stroke rehabilitation and if the control group received the same concomitant treatments as the acupuncture group. A randomization sequence generated from a random number table, calculator or computerized random number generator was considered authentic.

#### Types of participants

Patients of any gender, sex or ethnicity with ischemic or hemorrhagic stroke in the subacute or chronic phases were eligible. Stroke must be diagnosed according to the World Health Organization definition (rapidly developed clinical signs of focal (or global) disturbances of cerebral function, lasting more than 24 hours or leading to death, with no other apparent cause than of vascular origin [17]; or magnetic resonance imaging (MRI) or confirmed by computerized tomography (CT ).

#### Types of interventions

Acupuncture therapy will be defined as body acupuncture, scalp acupuncture, electro-acupuncture, warm needling, or ear acupuncture, etc. Studies that compare different types of acupuncture were excluded. Placebo acupuncture refers to a needle attached to the skin surface (not penetrating the skin but at the same acupoints) [18]. Sham acupuncture refers to: 1) a needle placed in an area close to but not in the acupuncture points [18]; 2) subliminal skin electro stimulation via electrodes attached to the skin [19].

### Assessment of quality of reporting

#### Rating of overall reporting quality

An overall quality score (OQS) with 15 items from the CONSORT 2010 statement [20] and 17 items from the STRICTA [21,22] were used (Table 1). Each item was scored 1 if it was reported and 0 if it was not clearly, or definitely not stated. The CONSORT discussion section items were excluded because we considered them too subjective to evaluate.

**Table 1:** Overall quality of reporting rating using items from the CONSORT Statement (n = 15)

**Item**	**Criteria**	**Description**	**No. of positive trials**	**%**	**95% CI**	**Cohen’s** ***к*** **coefficient**	**95% CI**
1	“Randomized” in the title or abstract	Study identified as a randomized controlled in the title or abstract	15	100	-	1.00	-
2	Background	Adequate description of the scientific background and explanation of rationale	12	80	57 to 100	0.74	0.50 to 0.98
3	Trial design	Description of trial design (such as parallel, factorial) including allocation ratio	10	67	40 to 94	0.71	0.45 to 1.00
4	Participants	Description of the eligibility criteria for participants	13	87	67 to 100	0.78	0.40 to 1.00
5	Interventions	Details of the interventions intended for each group	11	73	48 to 99	0.82	0.70 to 1.00
6	Outcomes	Definition of primary (and secondary when appropriate) outcome measures	4	27	1 to 52	0.85	0.75 to 0.99
7	Sample size	Description of sample size calculation	2	13	−6 to 33	0.83	0.68 to 1.00
8	Randomization	Description of the method used to generate the random sequence	9	60	32 to 88	0.76	0.60 to 0.99
12	Statistical methods	Description of the statistical methods used to compare groups for primary outcomes, subgroup analyses, or adjusted analyses	11	73	48 to 99	0.73	0.40 to 1.00
13	Flow chart	Details on the flow of participants through each stage of the trials (No. of patients randomly assigned, receiving intended treatment, completing the protocol and analyzed)	5	33	6 to 60	0.84	0.68 to 1.00
14	Recruitment	Dates defining the periods of recruitment and follow-up	6	40	12 to 68	0.65	0.38 to 0.99
15	Baseline data	An outline of baseline demographic and clinical characteristics of each group	14	93	79 to 100	0.68	0.42 to 1.00
17	Outcomes and estimation	For each primary and secondary outcome, a summary of results for each group is given, and the estimated effect size and its precision (e.g. 95% CI)	4	27	1 to 52	0.75	0.52 to 1.00
18	Ancillary analyses	Clear statement of whether subgroup/adjusted analyses were prespecified or exploratory	3	20	−3 to 43	0.68	0.35 to 0.99
19	Harms	Description of all important adverse events in each group	5	33	6 to 60	0.85	0.72 to 1.00

#### Rating of key methodological items

Concealment of allocation, appropriate blinding (either practitioner, participant or assessor), and analysis according to intention-to-treat (ITT) principle are highly related to potential sources of systematic bias and thus distortion in the estimation of the effect. Consequently, these three important key methodological items from the CONSORT 2010 Statement have been assessed separately. We then developed three “yes”/”no” items, so that emphasis was placed on quality of reporting rather than adequacy of trial design. A combined key methodological index score (MIS) was calculated for each trial by combining the scores of these three factors (range, 0 to 3).

### Data extraction & analysis

Each article was reviewed by two independent investigators. They extracted useful information by modified CONSORT and STRICTA checklists (Table 1, 2 and 3). After both raters finished their review, we calculated Cohen’s *к*-statistics to assess agreement between two reviewers. Agreement was judged as poor if *к ≤* 0.20; fair if 0.20 lower than *к ≤* 0.40; moderate if 0.40 lower than *к ≤* 0.60; substantial if 0.60 lower than *к ≤* 0.80; good if *к* higher than 0.80; and perfect if *к =* 1. Discrepancies were reviewed in detail and subsequently settled by consensus.

**Table 2:** Overall quality of reporting rating using items from STRICTA (n = 15)

**Item**	**Criteria**	**Description**	**No. of positive trials**	**%**	**95% CI**	**Cohen’s** ***к*** **coefficient**	**95% CI**
1	Acupuncture rationale	(1a) Style of acupuncture (e.g., Traditional Chinese Medicine, Japanese, Korean, Western medical, Five Element, ear acupuncture, etc.)	12	80	57 to 100	0.65	0.38 to 0.98
		(1b) Reasoning for treatment provided, based on historical context, literature sources and/or consensus methods, with references where appropriate	12	80	57 to 100	0.78	0.60 to 1.00
		(1c) Extent to which treatment was varied	10	67	40 to 94	0.72	0.48 to 0.95
2	Details of needling	(2a) Number of needle insertions per subject per session (mean and range where relevant)	10	67	40 to 94	0.75	0.60 to 0.99
		(2b) Names (or location if no standard name) of points used (uni-/bilateral)	12	80	57 to 100	0.83	0.65 to 1.00
		(2c) Depth of insertion, based on a specified unit of measurement or on a particular tissue level	8	53	25 to 82	0.92	0.81 to 1.00
		(2d) Responses sought (e.g., de qi or muscle twitch response)	9	60	32 to 88	0.63	0.38 to 1.00
		(2e) Needle stimulation (e.g., manual or electrical)	13	87	67 to 100	0.74	0.50 to 0.99
		(2f) Needle retention time	14	93	79 to 100	0.78	0.62 to 1.00
		(2 g)Needle type (diameter, length and manufacturer or material)	10	67	40 to 94	0.70	0.53 to 1.00
3	Treatment regimen	(3a) Number of treatment sessions	10	67	40 to 94	0.62	0.33 to 0.98
		(3b) Frequency and duration of treatment sessions	10	67	40 to 94	0.69	0.43 to 1.00
4	Other components of treatment	(4a) Details of other interventions administered to the acupuncture group (e.g., moxibustion, cupping, herbs, exercises, lifestyle advice)	3	20	−3 to 43	0.85	0.68 to 0.99
		(4b) Setting and context of treatment, including instructions to practitioners, and information and explanations to patients	5	33	6 to 60	0.64	0.35 to 0.98
5	Practitioner background	(5) Description of participating acupuncturists (qualification or professional affiliation, years in acupuncture practice, other relevant experience)	8	53	25 to 82	0.76	0.62 to 1.00
6	Control or comparator interventions	(6a) Rationale for the control or comparator in the context of the research question, with sources that justify the choice(s)	9	60	32 to 88	0.78	0.65 to 1.00
		(6b) Precise description of the control or comparator. If sham acupuncture or any other type of acupuncture-like control is used, provide details as for items 1–3 above	9	60	32 to 88	0.73	0.56 to 1.00

**Table 3:** Reporting quality of key methodological items (n = 15)

**Item**	**Criteria**	**Description**	**No. of positive trials**	**%**	**95% CI**	**Cohen’s** ***к*** **coefficient**	**95% CI**
9	Allocation concealment	Description of the method used to implement the random allocation sequence assuring the concealment until interventions are assigned	5	33	6 to 60	0.68	0.45 to 1.00
11	Blinding	Whether or not participants, those administering the interventions, or those assessing the outcomes were blinded to group assignment	9	60	32 to 88	0.75	0.52 to 1.00
16	Intent-to-treat analysis	No. of participants in each group included in each analysis and whether it was done by “intention to treat”	5	33	6 to 60	0.82	0.68 to 1.00

To identify factors associated with the overall quality of publications, we used the OQS as the outcome variable which was modeled using linear regression. Only variables that were significant at *P ≤* 0.10 in the univariate models were used in a multivariate regression model for selecting significant variables (*P ≤* 0.05). To identify factors associated with methodological quality, we used the MIS as the outcome variable in the regression analyses. As the outcome variable can be considered as a count, we relied on ordinal regression model and adjusted the variance empirically.

Linear and ordinal regression analysis was performed using SPSS version 20.0. Database of RCTs in acupuncture for stroke rehabilitation are provided in Additional file 1 and 2.

## Results

**Figure 1:**
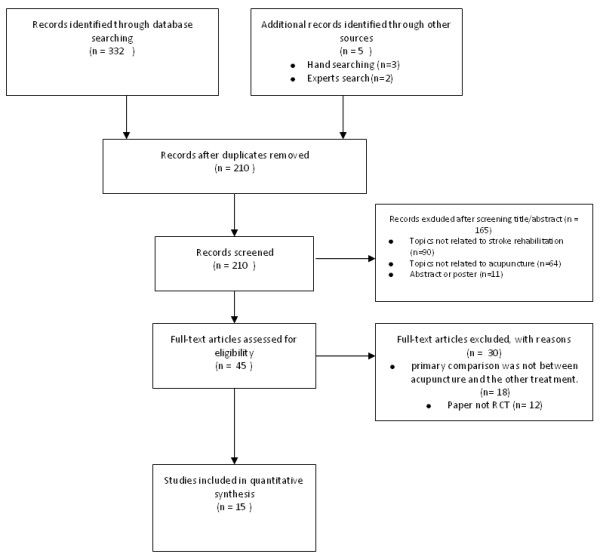
Diagram flow of the randomized controlled trials articles selection process.

The RCTs selection process is outlined in Figure 1. After identification, screening, eligibility determining of the literatures, a total of 15 relevant RCTs were included in the final analysis.

### Characteristics of the included trials

#### Year distribution of publication

Counting the number of articles, frequency, which refers to acupuncture for stroke rehabilitation in RCTs, was found to increase over time: from 2 (13.33%) in 1997–2001 to 8 (53.33%) in 2007-December, 2012.

#### Publication language

Of the 15 RCT papers, 10 (66.67%) were written in English. The remaining 5 papers (33.33%) were published in Chinese.

#### Nationality of authors

A total of 7 (46.67%) included papers were written by authors in Chinese research institutes; 7 (46.67%) were reported by international researchers; the remaining one paper (6.66%) was collaborations of Chinese and international researchers.

#### Funding source

Seven articles (46.67%) reported their sources of funding. Funding was obtained from provincial/municipal or international institutes.

#### Choice of comparator interventions

Interventions of nine trials (60.0%) comprised of sole acupuncture, followed by acupuncture plus other therapies (6 reports; 40.0%).

### Quality of reporting

#### Rating of overall reporting quality

The ratings of overall quality of reporting based on the CONSORT statement were listed in Table 1. When the 15 RCTs were considered, the median OQS was 8, with a minimum of 1 and a maximum of 15. Poor reporting existed in terms of “outcomes”, “sample size”, “outcomes and estimation”, “ancillary analyses” with positive rate of less than 30% (Table 1). All of the items had a substantial, good or perfect agreement (Table 1).

The ratings of overall quality of reporting based on STRICTA were listed in Table 2. When the 15 RCTs were considered, the median OQS was 12, with a minimum of 3 and a maximum of 16. Poor reporting existed in terms of “4a: Details of other interventions” and “4b: Setting and context of treatment” with positive rate of 20% and 33% (Table 2). All of the items had a substantial or good agreement (Table 2).

#### Rating of key methodological items

Allocation concealment, blinding, and analysis by intent to treat were reported in 5 (33%), 9 (60%), and 5 (33%) of the 15 RCTs, respectively (Table 3). The median MIS was 1 with a minimum of 0 and a maximum of 3 (Table 3). Among the 15 studies, 5 (33%) did not report any of the three key methodological items. All of the items had a substantial or good agreement (Table 3).

#### Exploratory analysis: factors associated with better reporting quality

In univariate analyses, nationality of authors and funding source were associated with an increased OQS based on the CONSORT statement. After adjustment, the multivariate linear regression model suggested that funding source remained independent and significant predictors of overall quality. Specifically, the mean OQS based on the CONSORT statement increased by about 4.05 for manuscripts with funding source (95% CI, 0.44 to 7.67; *P* < 0.05) (Table 4).

**Table 4:** Multivariate linear regression analysis for factors associated with better OQS from the CONSORT statement (n = 15)

**Variables**	***β***	**S.E.**	***t***	***P***	**95% CI**
Constant	2.32	2.59	0.90	0.39	−3.28 ~ 7.92
Funding source	4.05	1.67	2.43	0.03	0.44 ~ 7.67

In univariate analyses, only year of publication was associated with an increased OQS based on STRICTA. Specifically, the mean OQS based on STRICTA increased by about 0.48 for manuscripts published in the period of one year increment (95%CI, 0.00 ~ 0.96; *P* ≤ 0.05) (Table 5).

**Table 5:** Univariate analysis for factors associated with better OQS from STRICTA (n = 15)

**Variables**	***β***	**S.E.**	***t***	***P***	**95% CI**
Constant	−958.16	446.54	−2.15	0.05	−1922.84 ~ 6.53
Year of publication	0.48	0.22	2.17	0.05	0.00 ~ 0.96

With regard to the MIS, in ordinal regression analyses, no variable was associated with improved methodological quality (*P* > 0.05).

## Discussion

To the best of our knowledge, this study is the first investigation on the quality of reporting on RCTs of acupuncture for stroke rehabilitation according to the revised CONSORT and STRICTA guidelines. We found evidence that quality of reporting of the overall CONSORT items was general with median OQS 8 which was more than half of its total score 15; quality of reporting of the STRICTA items was good with median OQS 12 which was more than 70% of its total score 17; however, it is poor quality for the reporting of key methodological items with median MIS 1 which was 33% of its total score. Similar results were reported in some published studies [23,24]. Some studies found that the quality of reporting in RCTs on Chinese medicine remained poor (mainly in key methodological items), but had improved over time [25,26]. None of the variables we looked at in our ordinal regression model were significant predictors of improved methodological quality. We noted that the overall OQS based on the CONSORT Statement was correlated with funding source and STRICTA score was correlated with year of publication.

The purpose of reporting guidelines such as CONSORT and STRICTA is to increase the transparency of study methods and ultimately improve the overall quality of research. However, poorly reported trials make it ambiguous for other readers to assess the validity of the results and may mislead medical policy-makers in their decisions. Our study found that the overall quality of reporting was general or good between 1997 to March 2013. There might be multiple reasons. First, most of included reports are published after 2001 when STRICTA and revised CONSORT statement was published, especially, STRICTA score was correlated with year of publication. In the evaluation of acupuncture trials performed by Prady et al. [27], the authors concluded that reporting of CONSORT items had improved after the introduction of CONSORT but that the introduction of STRICTA did not improve the reporting of STRICTA items. However, another evaluation of auriculotherapy trials [23] came to similar conclusions with ours. Maybe the introduction of STRICTA and CONSORT statement improved the overall quality of reporting in our research. Second, more and more clinicians were training in study design and reporting of RCTs, and some better quality research papers had been submitted and published in international journals which had a more rigorous peer-review process. Our finding that the overall OQS based on the CONSORT statement was correlated with funding source, also suggested clinical trials with funding have more capacity to provide assurance for the better quality of study design and reporting of RCTs.

However, some items’ reporting was found where information was insufficient or inadequate in most studies. These areas are as follows: “allocation concealment”, “analysis by intent to treat”, “outcomes”, “sample size”, “outcomes and estimation“ and “ancillary analyses” based on the CONSORT statement; “details of other interventions” and “setting and context of treatment” based on STRICTA.

A generated allocation schedule should be implemented by using allocation concealment, a critical mechanism that prevents foreknowledge of treatment assignment and thus shields those who enroll participants from being influenced by this knowledge [20]. Trials in which the allocation sequence had been inadequately or unclearly concealed yielded larger estimates of treatment effects than did trials in which authors reported adequate allocation concealment. Intention to treat was defined as the inclusion of all patients randomly assigned in the analysis, regardless of whether they actually satisfied the entry criteria, the treatment actually received, and subsequent withdrawal or protocol deviations [25,27]. ITT is generally favored because it avoids bias associated with non-random loss of participants. Unfortunately, we found only 33% of the trial reports provided a description of ITT or allocation concealment, making it very difficult to judge the validity of their findings.

Authors should indicate how the sample size and primary outcome was determined. Theoretically, clinical significant difference between interventions was detected by a high power if a trial had enough number of subjects. The researcher should note the primary and second outcome on which the calculation was based. For each primary and secondary outcome, results for each group, the estimated effect size and its precision (e.g. 95% confidence interval) presentation were recommended. However, no more than 30% of included reports presented the primary and second outcome, sample size calculation and outcome estimation, etc.

As for STRICTA, “details of other interventions” referred to the auxiliary techniques, prescribed self-treatment and lifestyle advice provided by the practitioner. All additional components, whether carried out by the practitioner or patient and whether integral or adjunctive to the acupuncture needling, should be described clearly [21]. “Setting and context of treatment” could also provide important additional components to treatment. For patients, the context factor includes some information given by practitioners might modify the trial’s outcome; for practitioners, it includes some instructions given by patients after prescribing or proscribing explanations to them might modify practitioners’ normal practice [21]. But the number of reports provided this information was small. As different acupuncturists providing treatment to different treatment arms will influence generalisability of the trial results, the details of other interventions and the background of both groups should be explained.

One limitation we should point out is that, due to language barrier, we didn’t search for any manuscripts published in non- Chinese or English journals. This is an area for worthwhile future study. It remains unknown whether searching in other language journals would have altered the constitution of our sample or results.

## Conclusions

Our study found that the overall quality of reporting on RCTs of acupuncture for stroke rehabilitation was general or good. however, it is poor quality for the reporting of key methodological items. our results stress the need for researchers involved in RCTs of acupucture for stroke to improve the methodological quality of their research through the introduction of STRICTA and CONSORT statement.

## Competing interests

The authors declare that they have no conflicts of interest concerning this article.

## Authors’ contributions

ZLX was responsible for conception and design of the review, carried out the literature search, performed data analysis, and drafted the manuscript. HJ and ZX performed data extraction and assessment of risk of bias, participated in conception and design of the review, and critically revised the man ± uscript. LLM participated in conception and design of the review, and critically revised the manuscript. All authors read and approved the final manuscript.

## Pre-publication history

The pre-publication history for this paper can be accessed here:

http://www.biomedcentral.com/1472-6882/14/151/prepub

## Supplementary Material

Additional file 1 CONSORT evaluation of acupuncture for stroke rehabilitation. Click here for file

Additional file 2 STRICTA evaluation of acupuncture for stroke rehabilitation. Click here for file

## References

[B1] Mathers CD Boerma T Ma Fat D Global and regional causes of death Br Med Bull 2009 92 7 32 10.1093/bmb/ldp028 19776034

[B2] Liu L Wang D Wong KS Wang Y Stroke and stroke care in China: huge burden, significant workload, and a national priority Stroke 2011 42 12 3651 3654 10.1161/STROKEAHA.111.635755 22052510

[B3] American Heart Association Update at-a-Glance, Heart Disease and Stroke Statistics Dallas: Am Heart Assoc 2009 2009 14 16

[B4] American Heart Association Update, Heart Disease and Stroke Statistics Dallas: Am Heart Assoc 2004 2004 13 17

[B5] Barnes PM Bloom B Nahin RL Complementary and alternative medicine use among adults and children: United States. 2007 Nati Health Stat Report 2008 10 12 1 23 19361005

[B6] Sze FK Wong E Or KK Woo J Does acupuncture improve motor recovery after stroke? A meta-analysis of randomized controlled trials Stroke 2002 33 11 2604 2619 10.1161/01.STR.0000035908.74261.C9 12411650

[B7] Park J Hopwood V White AR Ernst E Effectiveness of acupuncture for stroke: a systematic review J Neurol 2001 248 7 558 563 10.1007/s004150170132 11517996

[B8] Kong JC Lee MS Shin BC Song YS Ernst E Acupuncttire for functional recovery after stroke: a systematic review of sham-controlled randomized clinical trials CMAJ 2010 182 16 1723 1729 10.1503/cmaj.091113 20876268 PMC2972322

[B9] Wu HM Tang JL Lin XP Lau J Leung PC Woo J Li YP Acupuncture for stroke rehabilitation (Review) 2009 Library: The Cochrane doi:10.1002/14651858.CD004131.pub2 10.1002/14651858.CD004131.pub2 16856031

[B10] Pei J Sun L Chen R Zhu T Qian Y Yuan D The effect of electro-acupuncture on motor function recovery in patients with acute cerebral infarction: a randomly controlled trial J Tradit Chin Med 2001 21 4 270 272 12014128

[B11] Sällström S Kjendahl A Osten PE Stanghelle JK Borchgrevink CF Acupuncture therapy in stroke during the subacute phase. A randomized controlled trial [Article in Norwegian] Tidsskr Nor Laegeforen 1995 115 23 2884 2887 7570509

[B12] Si QM Wu GC Cao XD Effects of electroacupuncture on acute cerebral infarction Acupunct Electrother Res 1998 23 2 117 124 9789586 10.3727/036012998816356562

[B13] Wu P Mills E Moher D Seely D Acupuncture in poststroke rehabilitation: a systematic review and meta-analysis of randomized trials Stroke 2010 41 4 el71 el79 10.1161/STROKEAHA.109.573576 20167912

[B14] Begg C Cho M Eastwood S Horton R Moher D Olkin I Pitkin R Rennie D Schulz KF Simel D Stroup DF Improving the quality of reporting of randomized controlled trials The CONSORT statement JAMA 1996 276 8 637 639 10.1001/jama.276.8.637 8773637

[B15] MacPherson H Altman DG Hammerschlag R Revised STandards for Reporting Interventions in Clinical Trials of Acupuncture (STRICTA): Extending the CONSORT statement J Evid Based Med 2010 3 3 140 155 10.1111/j.1756-5391.2010.01086.x 21349059

[B16] Hugh M Altman DG Hammerschlag R Hammerschlag R Li YP Wu TX White A Moher D Revised STandards for Reporting Interventions in Clinical Trials of Acupuncture (STRICTA): extending the CONSORT statement Zhong Xi Yi Jie He Xue Bao 2010 8 9 804 818 20836969 10.3736/jcim20100902

[B17] Asplund K Tuomilehto J Stegmayr B Wester PO Tunstall-Pedoe H Diagnostic criteria and quality control of the registration of stroke events in the MONICA project Acta Med Scand Suppl 1988 728 26 39 3202029 10.1111/j.0954-6820.1988.tb05550.x

[B18] Tulder MW VA Cherkin DC Berman B Lao L Koes BW Acupuncture for low back pain Cochrane Database Syst Rev 2000 2 CD001351 10.1002/14651858.CD001351 10796434

[B19] Swedish Collaboration on Sensory Stimulation in Stroke Sensory stimulation after stroke: a randomized controlled trial Cerebrovasc Dis 1999 9 Suppl 1 289873160

[B20] Moher D Hopewell S Schulz KF Montori V Gøtzsche PC Devereaux PJ Elbourne D Egger M Altman DG CONSORT 2010 Explanation and Elaboration: updated guidelines for reporting parallel group randomised trials BMJ 2010 340 c869 10.1136/bmj.c869 20332511 PMC2844943

[B21] Baer H **The emergence of integrative medicine in Australia: the growing interest of biomedicine and nursing in complementary medicine in a southern developed society** Med Anthropol Q 2008 22 1 52 66 10.1111/j.1548-1387.2008.00003.x 18610813

[B22] Prady SL Richmond SJ Morton VM Macpherson H A systematic evaluation of the impact of STRICTA and CONSORT recommendations on quality of reporting for acupuncture trials PLoS One 2008 3 2 e1577 10.1371/journal.pone.0001577 18270568 PMC2216683

[B23] Asher GN Motsinger-Reif AA Jonas DE Viera AJ Quality of reporting on randomised controlled trials of auriculotherapy for pain Acupunct Med 2011 29 2 122 126 10.1136/aim.2010.003475 21487067

[B24] Hammerschlag R Milley R Colbert A Weih J Yohalem-Ilsley B Mist S Aickin M Randomized Controlled Trials of Acupuncture (1997–2007): An Assessment of Reporting Quality with a CONSORT- and STRICTA-Based Instrument Evid Based Complement Alternat Med 2011 doi:10.1155/2011/183910 10.1155/2011/183910 PMC2952291 20953418

[B25] Wang G Mao B Xiong ZY Fan T Chen XD Wang L Liu GJ Liu J Guo J Chang J Wu TX Li TQ CONSORT Group for Traditional Chinese Medicine The quality of reporting of randomized controlled trials of traditional Chinese medicine: a survey of 13 randomly selected journals from mainland China Clin Ther 2007 29 7 1456 1467 10.1016/j.clinthera.2007.07.023 17825697

[B26] Wang L Li Y Li J Zhang M Xu L Yuan W Wang G Hopewell S Quality of reporting of trial abstracts needs to be improved: using the CONSORT for abstracts to assess the four leading Chinese medical journals of traditional Chinese medicine Trials 2010 11 75 doi:10.1186/1745-6215-11-75 10.1186/1745-6215-11-75 20615225 PMC2911423

[B27] Toulmonde M Bellera C Mathoulin-Pelissier S Debled M Bui B Italiano A Quality of randomized controlled trials reporting in the treatment of sarcomas J Clin Oncol 2011 29 9 1204 1209 10.1200/JCO.2010.30.9369 21321290

